# Comparative Study on the Phytochemical Characterization and Biological Activities of *Azolla caroliniana* and *Azolla filiculoides*: In Vitro Study

**DOI:** 10.3390/plants12183229

**Published:** 2023-09-11

**Authors:** Salwa M. Abdel Rahman, Maher A. Kamel, Mennatallah A. Ali, Badriyah S. Alotaibi, Ohud Muslat Aharthy, Mustafa Shukry, Hala Mohamed Abd El-Bary

**Affiliations:** 1 Botany and Microbiology Department, Faculty of Science, Alexandria University, Alexandria 21511, Egypt; 2 Department of Biochemistry, Medical Research Institute, Alexandria University, Alexandria 21516, Egypt; 3 Department of Pharmacology and Therapeutics, Faculty of Pharmacy, Pharos University in Alexandria, Alexandria 21544, Egypt; 4 Department of Pharmaceutical Sciences, College of Pharmacy, Princess Nourah bint Abdulrahman University, P.O. Box 84428, Riyadh 11671, Saudi Arabia; 5 Department of Biotechnology, Faculty of Science, Taif University, P.O. Box 11099, Taif 21944, Saudi Arabia; 6 Physiology Department, Faculty of Veterinary Medicine, Kafrelsheikh University, Kafrelsheikh 33516, Egypt

**Keywords:** *Azolla caroliniana*, *Azolla filiculoides*, antioxidant activity, anti-inflammatory activity, cytotoxicity

## Abstract

Azolla is a floating fern known for its various biological activities. *Azolla caroliniana* and *Azolla filiculoides* are multifunctional plants that exhibit biological activity in multiple ways, making them beneficial for various applications. This study aimed to compare the phytochemical composition and antimicrobial, antioxidant, anti-inflammatory, and cytotoxicity activities of two Azolla species, namely *Azolla caroliniana* and *Azolla filiculoides*. GC-MS analysis revealed distinct patterns of phytochemical composition in the two species. The methanol extracts of *A. caroliniana* and *A. filiculoides* exhibited moderate antimicrobial activity against *Geotrichum candidum*, *Enterococcus faecalis*, and *Klebsiella pneumonia*. Furthermore, both extracts demonstrated potential antioxidant activity, as evidenced by a dose-dependent increase in a ferric-reducing activity power (FRAP) assay. Additionally, the extracts showed promising anti-inflammatory activities, including inhibition of protein denaturation, heat-induced red blood cell (RBC) hemolysis, and nitric oxide (NO) production by macrophages. Moreover, the methanolic extracts of *A. caroliniana* displayed higher cytotoxicity against HepG2 cells than those of *A. filiculoides* in a dose-dependent manner. These findings suggest that the methanolic extracts of *A. caroliniana* and *A. filiculoides* contain distinct compounds and exhibit potential antioxidant, anti-inflammatory, and cytotoxic activities against HepG2 cells. In conclusion, our data indicate that the methanolic extracts of *A. caroliniana* and *A. filiculoides* have differential phytochemical compositions and possess potential antioxidant, anti-inflammatory, and HepG2 cytotoxic activities.

## 1. Introduction

Azolla is a plant found in the tropics, subtropics, and warm temperate regions. It floats on the water with small, fern-like leaves [1]. Pteridophyte Anabaena *azollae* is symbiotic with this floating plant [2]. This host–symbiont combination is exploited as a biofertilizer for many crops [3]. In northern Vietnam and central and southern China, Azolla has been utilized as green manure for wetland rice for millennia [4]. In addition, feeding Azolla to dairy cattle, pigs, ducks, and chickens reportedly increases milk production, pig weight, and chicken egg production [4]. Azolla meal prepared from *Azolla pinnata* was reported to provide optimal growth in cultured shrimp [5]. In addition, Azolla is suggested as part of a space diet when living on Mars as an essential vegetarian diet adequate for human health [6].

The species in the genus Azolla are divided into two subgenera. The subgenus Azolla (Euazolla) contains four species, all of which are endemic to America: *A. Carolina*, *A. mexicana*, *A. microphylla*, and *A. filiculoides*. In contrast, the *A. nilotica* and the *A. pinnata* species of the subgenus Rihizosperma are endemic to Africa [7]. Eastern North America and the Caribbean are home to *Azolla caroliniana*, while southern South America, western North America, and Alaska are the distribution ranges for *A. filiculoides* [8]. 

Azolla’s beneficial phytochemicals include flavonoids, hormones, alkaloids, phenols, triterpenoid derivatives, amino acids, and fatty acids. Antioxidant, anticarcinogenic, anti-inflammatory, antidiabetic, hepato- and gastroprotective, antiviral, neuroprotective, cardioprotective, and antihypertensive effects can all be attributed to these bioactive components [9].

Although many articles have been written on the biology, uses, and factors altering the growth and phytochemistry of species of Azolla and their biological roles [10], few studies have discussed the phytochemical and biological screening of *Azolla caroliniana* and *Azolla filiculoides* [11]. In the present study, we compared the phytochemical composition, antimicrobial activity, antioxidant activity, anti-inflammatory activity, and cytotoxicity activity of two Azolla species, namely *Azolla caroliniana* and *Azolla filiculoides*.

## 2. Results

This study was conducted on *Azolla caroliniana* (C) and *Azolla filiculoides* (F) and involved phytochemical screening, as well as comparison of their antimicrobial, total antioxidant, anti-inflammatory, and cytotoxicity activities.

### 2.1. Phytochemical Screening

**Figure 1:**
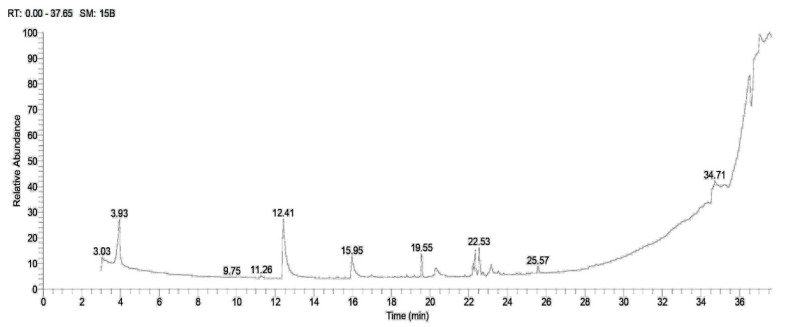
GC–MS chromatogram of methanolic extract of *A. caroliniana*.

**Figure 2:**
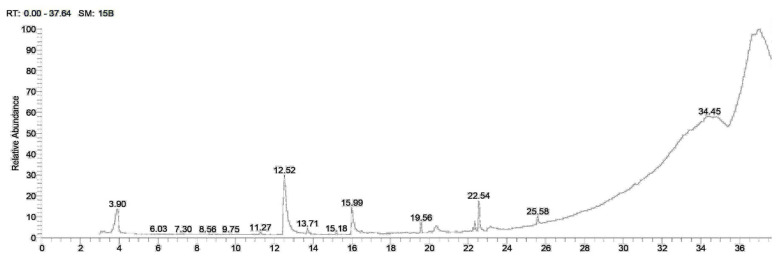
GC–MS chromatogram of methanolic extract of *A. filiculoides*.

Methanol extracts of *A. caroliniana* and *A. filiculoides* were analyzed using GC-MS; the findings are presented in Table 1. Figure 1 and Figure 2 display the chromatograms of total ions. The retention times of the peaks ranged from 3.03 to 36.49. The amounts of phytochemicals found in the samples are presented as a proportion of the total extract (area percent). Quantitative variations in the content of certain phytochemicals were observed between *A. caroliniana* and *A. filiculoides*. In the methanol extract of *A. caroliniana*, the percentage of dimethyl lauramine was 13.11, while in *A. filiculoides*, it was 23.89. Similarly, the percentage of ethyl linoleate was 2.26 in *A. caroliniana* and 1.14 in *A. filiculoides*. The methanol extract of *A. caroliniana* contained cyclopropane butanoic acid, 2-[[2-[[2-[(2-pentylcyclopropyl) methyl]cyclopropyl]methyl]cyclopropyl]methyl]-, and methyl ester, whereas isochiapin B was detected in the methanol extract of *A. filiculoides*.

Furthermore, the percentages of N-Methyl-N-benzyl, tetradecanamine, and trilinolein in the methanol extract of *A. filiculoides* were approximately 1.5, 1.7, and 1.6-fold higher, respectively, than in the methanol extract of *A. caroliniana*.

### 2.2. Antimicrobial Activity

Table 2 shows the inhibition diameters of agar plates treated with methanol extracts of *A. caroliniana* and *A. filiculoides* for our four fungal strains (Gram-positive and Gram-negative). All tested fungi were resistant to methanol extracts of *A. caroliniana* and *A. filiculoides*, except *Geotrichum candidum,* which showed sensitivity toward both extracts, especially toward *A. caroliniana* extract, which produced a larger zone of inhibition than *A. filiculoides* extract, although the difference was not significant. On the other hand, *Enterococcus faecalis* and *Klebsiella pneumonia* showed moderate sensitivity toward the methanolic extracts of Azolla species.

The antimicrobial activity is presented in Table 3 as the minimum inhibitory concentration (MIC) of the tested microorganisms in μg/mL. The MIC was defined as the minimum concentration that resulted in no growth. The methanol extract of *A. caroliniana* has superior antimicrobial activity against *Geotrichum candidum* and *Enterococcus faecalis* compared to that of *A. filiculoides*, with MIC values of 312.5 and 625 μg/mL, respectively.

### 2.3. Ferric-Reducing Antioxidant Power

**Figure 3:**
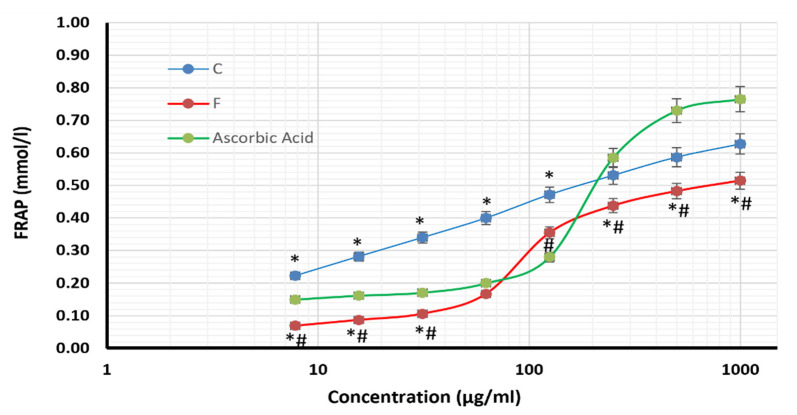
Ferric-reducing antioxidant power of the methanolic extract of *A. caroliniana* (C), *filiculoides* (F), and ascorbic acid. * Significant difference compared with the same dose of ascorbic acid; # significant difference compared with the same dose of C according to by ANOVA at *p* < 0.05.

The antioxidant abilities of the methanolic extracts from the two Azolla species were evaluated; the results are presented in Table 4 and Figure 3. Both extracts demonstrated antioxidant power, as evidenced by increasing ferric-reducing antioxidant power (FRAP) with higher concentrations. However, the methanolic extract of *A. caroliniana* exhibited more robust FRAP activity than the extract of *A. filiculoides* and ascorbic acid. The IC_50_ values for *A. caroliniana*, *A. filiculoides*, and ascorbic acid were 55.86 µg/mL, 92.78 µg/mL, and 416.2 µg/mL, respectively.

### 2.4. Anti-Inflammatory Activity

**Figure 4:**
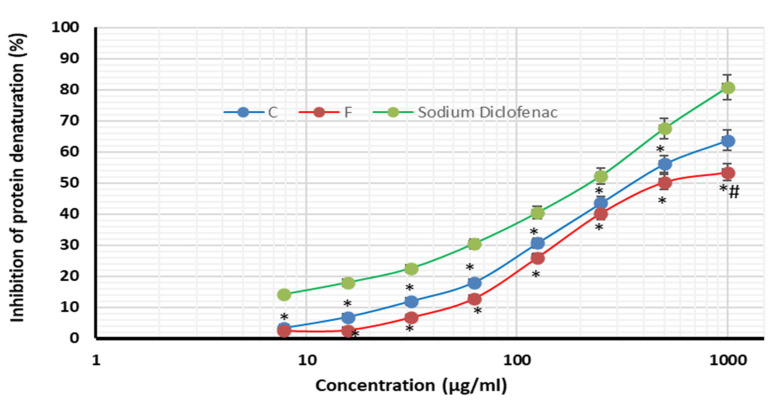
Inhibition of protein denaturation by the methanolic extracts of *A. caroliniana* (C), *filiculoides* (F), and sodium diclofenac. * Significant difference compared with the same dose of sodium diclofenac; # significant difference compared with the same dose of C according to ANOVA at *p* < 0.05.

**Figure 5:**
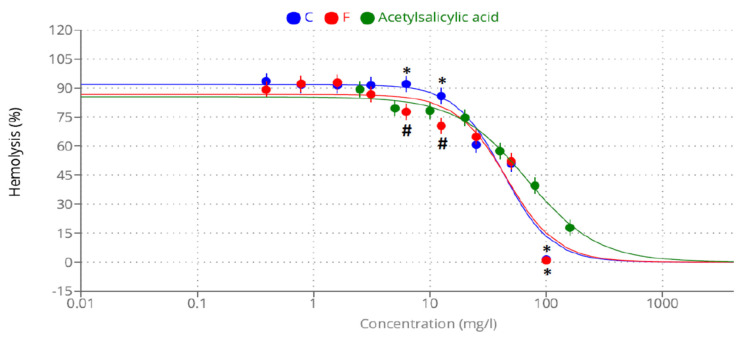
The inhibition of heat-induced hemolysis of methanolic extracts of *A. caroliniana* (C), *filiculoides* (F), and acetylsalicylic acid. * Significant difference compared with the same dose of acetylsalicylic acid; # significant difference compared with the same dose of C according to ANOVA at *p* < 0.05.

**Figure 6:**
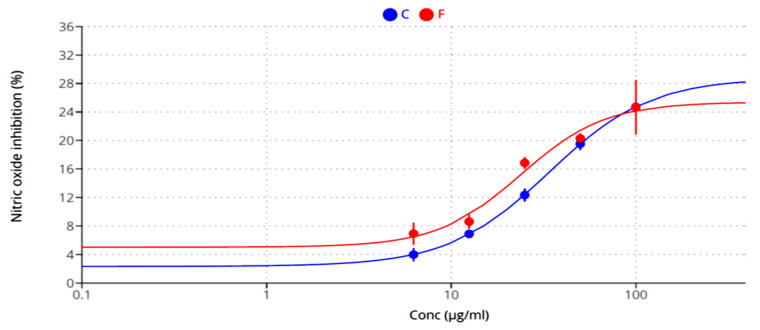
The inhibition of nitric oxide production by methanolic extracts of *A. caroliniana* (C) and *filiculoides* (F).

Figure 4, Figure 5 and Figure 6 highlight the extracts’ anti-inflammatory activities, including their ability to prevent protein denaturation and heat-induced hemolysis while also blocking NO production.

### 2.5. Cytotoxicity Activity

**Figure 7:**
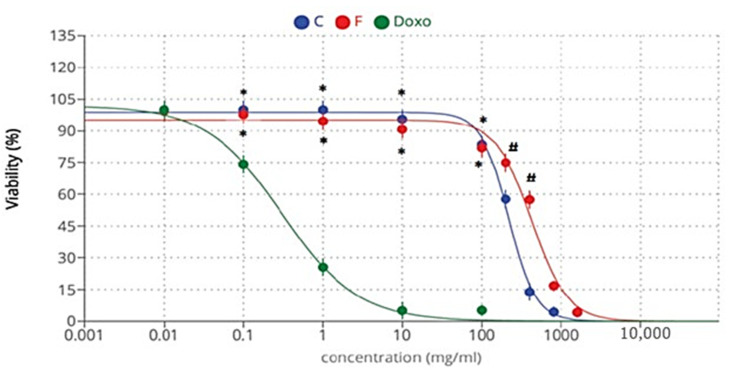
Cytotoxicity of methanolic extracts of *A. caroliniana* (C) and *filiculoides* (F), as well as doxorubicin (Doxo), against the HepG2 cell line. * Significant difference compared with the same dose of doxorubicin; # significant difference compared with the same dose of C according to ANOVA at *p* < 0.05).

This experiment examines the impacts of the methanol extracts of *A. caroliniana* and *A. filiculoides* on the HepG2 cell line. The methanolic extracts *A. caroliniana* and *A. filiculoides* showed similar cytotoxicity against the HepG2 cell line only at higher doses compared with the standard drug used. The cytotoxicity of doxorubicin (Figure 7) with the IC_50_ of *A. caroliniana* was 216.86 µg/mL, that of *A. filiculoides* was 421.2 µg/mL, and that of doxorubicin was 0.303 µg/mL (Table 4). These results indicate the more efficient antiproliferative effect of *A. caroliniana* relative to that of *A. filiculoides* methanol extract against the HepG2 cells.

## 3. Discussion

Aquatic ferns of the genus Azolla are harvested for their usefulness as a biofertilizer and a source of bioactive chemicals. In this study, we compared the chemical composition and biological activities of methanolic extracts from two of the most investigated species, *Azolla caroliniana* and *Azolla filiculoides*.

The two species showed a differential pattern of phytochemical compositions; the methanolic extract of *A. caroliniana* is more prosperous than the extract of *A. filiculoides* in in terms of the contents of 9-octadecenoic acid, (2-phenyl-1,3-dioxolan-4-yl) methyl ester, cis-, methyl palmitate, ethyl linoleate, 9,12-octadecadienoic acid (Z, Z)-,2-hydroxy-1-(hydroxymethyl)ethyl ester, and nicotiflorin. However, the concentrations of isochiapin B, dimethyl lauramine, n-methyl-N-benzyl tetradecanamine, and trilinolein are more significant in the extract of *A. filiculoides*. The two extracts contain equivalent amounts of the remaining ingredients, such as calcitriol, rhodopin, pentadecanoic acid, tricyclo [20.8.0.0(7,16)] triacontane, and 1(22),7(16)-diepoxy.

The methanol extracts of *A. caroliniana* and *A. filiculoides* exhibited moderate antimicrobial activity against *Geotrichum candidum*, *Enterococcus faecalis*, and *Klebsiella pneumonia*. These results are consistent with those of a prior study by Sathammaipriya et al. [12], which indicated that *Azolla microphylla* leaf extracts possess good antimicrobial activity versus *Bacillus* sp., *Staphylococcus* sp., *Escherichia coli*, *Klebsiella* sp., and *Proteus* sp. Synergistic interactions between several phytochemicals found in methanol extracts of *A. caroliniana* and *A. filiculoides* may be responsible for their antibacterial activity. Previous reports indicated that 9-octadecenoic acid, (2-phenyl-1,3-dioxolan-4-yl) methyl ester, cis- [13], methyl palmitate [14], ethyl linoleate [15], isochiapin B [16], and calcitriol [17], in addition to aliphatic chains, hydroxyl groups, and fatty acids [18], exhibited antimicrobial activity.

2-[[2-[[2-[(2-pentyl cyclopropyl) methyl] cyclopropyl]methyl]cyclopropanebutanoic acid methyl ester is a compound that has been extensively studied, with documented effects on anti-inflammatory, antibacterial, antifungal, and skin-conditioning activities. These properties make it a valuable compound with potential applications in various fields [19].

The methanolic extract of *A. caroliniana* showed potential antioxidants activity as indicated by a dose-dependent increase in an FRAP assay and low a IC_50_ (55.86 µg/mL), which is more potent than that of ascorbic acid (IC_50_ = 416.21 µg/mL). The methanolic extract of *A. filiculoides* achieve no or a mild increase in FRAP at low doses (up to 125 µg/mL). At higher doses, the antioxidant power increased dose-dependently with IC_50_ at about 92.78 µg/mL. Furthermore, the ethanolic extract of *Azolla filiculoides* was found to have hepatoprotective and antioxidant activities [20].

Lu et al. [21] and Shahidi [22] proved that pigments such as chlorophylls, carotenoids, and vitamins; vitamin precursors such as phenol, carotene, niacin, thiamine, and ascorbic acid; and phenolic compounds such as polyphenols, hydroquinones, and flavonoids may be responsible for the antioxidant activity of marine algae. Chan et al. [23] showed that trilinolein exhibits an antioxidant effect. The antioxidant effect may also be credited to antioxidants and immune-boosting phytocontents (vitamins and phytochemicals including carotenoids, flavonoids, and tannins) [24]. Azolla’s high carotene, phenolic, and flavonoid contents are also credited for its immune-enhancing effects [25]. In line with these findings, Aron et al. [26] found that the antioxidant and anti-inflammatory actions of *A. pinnata*’s components may account for their protective benefits against lead-acetate-induced hepatotoxicity.

The methanolic extracts of *A. caroliniana* and *A. filiculoides* exhibited potential anti-inflammatory activities, protein stability, resistance to heat-induced RBC hemolysis, and macrophage NO generation, all aided by this compound. It is clear that the extracts of *A. caroliniana* have better anti-inflammatory activities, as indicated by lower values of IC_50_ in the protein denaturation assay (360 µg/mL) and inhibition of heat-induced RBC hemolysis (42.78 µg/mL) compared with the extracts of *A. filiculoides* is (IC_50_ = 527.3 and 45.85 µg/mL, respectively). On the contrary, the extract of *A. filiculoides* displayed better inhibition of NO production by macrophages (23.8 µg/mL) than that of *A. caroliniana* (33.92.8 µg/mL).

The methanol extract of *A. caroliniana* has superior anti-inflammatory activity to that of *A. filiculoides*. The mechanism of action of the methanolic extracts depends on their components chemicals that may be bound to the erythrocyte membranes, resulting in a change in the surface charges of the cells. However, the precise mechanism of membrane stabilization is still unknown. As a result, the hemolysis of red blood cells may spread more quickly due to the repulsion between like charges [27].

Due to their high contents of active ingredients, methanol extracts can induce anti-inflammatory activity through multiple pathways. The biological effects of extracts can be attributed to the presence of calcitriol; trilinolein; rhodopin; glycerides such as 9-Octadecenoic acid, (2-phenyl-1,3-dioxolan-4-yl) methyl ester, cis-, 9,12-octadecadienoic acid (Z, Z)-, 2-hydroxy-1-(hydroxymethyl)ethyl; ester and fatty acids and their derivatives, such as palmitic, linoleic, and pentadecanoic acids. Similar findings were reported in previous studies [28].

Calcitriol has been shown to decrease the expression of IL-17, INF-, and TNF- in T cells while simultaneously increasing the expression level of anti-inflammatory cytokine IL-10 [29]. Furthermore, trilinolein inhibited the protein expression of various proinflammatory mediators, including inducible nitric oxide synthase (iNOS), cyclooxygenase-2 (COX-2), nuclear factor-κB (NF-κB), κBα, TNF-α, IL-1β, IL-6, and mitogen-activated protein kinases [30].

Another important constituent of *A. caroliniana* and *A. filiculoides* that may induce anti-inflammatory activity is rhodopin (carotenoid), which prevents blood vessel growth by preventing transcription factor activation and nuclear translocation [31]. Azolla has been shown to have immune-enhancing effects due to its high concentrations of carotene, phenolic, and flavonoid components [25].

Another proposed explanation for the anti-inflammatory activity is methyl palmitate, a fatty acid ester found in nature that has been shown to prevent the production of inflammatory cells. Methyl palmitate ameliorated histopathological abnormalities and decreased plasma levels of tumor necrosis factor alpha and interleukin 6 in rats with endotoxemia produced by lipopolysaccharide (LPS) [32].

The severity of anemia, dyslipidemia, and fibrosis can be mitigated by the anti-inflammatory properties of pentadecanoic acid [33]. In addition, it stimulates alpha and delta peroxisome proliferator-activated receptors and restores mitochondrial activity [34]. Increased consumption of these odd-chain saturated fatty acids is associated with a lower risk of developing cardiovascular disease, obesity, chronic inflammation, type 2 diabetes, metabolic syndrome, fatty liver disease, emphysema, chronic obstructive pulmonary disease (COPD), and pancreatic cancer [35].

In addition, Park et al. [36] reported that in LPS-activated RAW 264.7 cells, ethyl linoleate inhibits iNOS and COX-2 expression, decreasing NO and prostaglandin E2 synthesis. In addition, the heme oxygenase-1 (HO-1) small interfering (Si) RNA system confirmed that ethyl linoleate exhibited anti-inflammatory effects by increasing HO-1 expression. When HO-1 was knocked-down in LPS-induced macrophages using siRNA, the inhibitory effects of ethyl linoleate on NO, TNF-α, IL-1β, and IL-6 production were lost.

In this study, we examined the impacts of the methanolic extracts of *A. caroliniana* and *A. filiculoides* on hepatocellular carcinoma cell lines (HepG2). The results show that the methanolic extracts of *A. caroliniana* and *A. filiculoides* have significant cytotoxicity against HepG2 in a dose-dependent manner, with IC_50_ values of 216.86 and 421.20 µg/mL, respectively. The methanol crude extract of *A. caroliniana* had a higher cytotoxicity on the HepG-2 cell line than that of *A. filiculoides.*

Using GC-MS analysis, several substances with possible anticancer properties have been identified. Long-chain fatty acids and their derivatives have been found in extracts, including octadecadienoic acid [37], pentadecanoic acid [35], and methyl palmitate [38].

Elangovan et al. [39] showed the importance of vitamin D (calcitriol) and the vitamin D receptor axis in regulating the onset and severity of various hepatic lesions, including HCC. Rai V et al. [40] reported that vitamin D supplementation may help HCC by reducing inflammation and halting the course of the disease. Cultured leukemia cells but not normal cells responded to increasing concentrations of carotene by undergoing apoptosis and cell differentiation [41]. In another trial, carotene was shown to mitigate the harmful effects of radiation therapy, as well as the risk of cancer returning in the treated area [42]. Cytotoxic activities against hepatocarcinoma cell lines were observed for the Kei apple extract components isochiapin B, dotriacontane [16], and antioxidant pursuit [43], and antibacterial and antiviral actions [18] were recorded.

Multiple biological activities of the two Azolla species have been documented in previous research, as well as this investigation (*caroliniana*, and *filiculoides*). The potential uses of these chemicals in industries such as agriculture, health, and food science are only beginning to be explored.

## 4. Materials and Methods

### 4.1. Chemicals and Supplies

All chemicals used for the analysis were of analytically pure grade and purchased from (Sigma-Aldrich, St. Louis, MO, USA), including dimethyl sulfoxide (DMSO), tripyridyltriazine (TPTZ), ferric chloride (FeCl_3_), sodium citrate (Na-citrate), citric acid, ferrous sulfate heptahydrate (FeSO_4_∙7H_2_O), bovine serum albumin (BSA), sodium diclofenac, ascorbic acid, phosphate-buffered saline (PBS), acetylsalicylic acid, fetal bovine serum, penicillin, streptomycin sulfate, glutamine, lipopolysaccharides (LPS), Nω-nitro-L-arginine methyl ester hydrochloride (L-NAME), trichloroacetic acid (TCA), and tris(hydroxymethyl)aminomethane (TRIS).

The following strains were used in the tests: Fusarium *oxysporum* (RCMB 001004), Trichophyton *rubrum* (RCMB025002), Candida *albicans* RCMB 005003 (1) ATCC10231, *Geotrichum candidum* (RCMB 041001), *Staphylococcus aureus* ATCC25923, *Enterococcus faecalis* ATCC 29212, *Enterobacter cloacae* RCMB 001 (1) ATCC 23355, and *Klebsiella pneumonia* RCMB 003 (1) ATCC 13883, all obtained from the regional center for Mycology and Biotechnology, Al-Azhar University, Egypt. Nawah Scientific Inc. supplied the HepG2 hepatocellular carcinoma cells and RAW264.7 murine macrophages (ATCC^®^) used in this study (Mokatam, Cairo, Egypt).

### 4.2. Plant Material and Growth Conditions

Two different species of Azolla—*Azolla caroliniana* and *Azolla filiculoides*—were screened for phytochemical and biological activity. The two species were acclimatized in polyethylene vessels filled with nitrogen-free, modified (KCl and CaCl_2_ replaced with KNO_3_ and Ca (NO_3_)_2_, respectively) 2/5 concentration of Hoagland’s nutrition solution (pH 5.6) at the greenhouse of the Faculty of Science Alexandria University. Each vessel was inoculated with about 5 g of Azolla from the stock material to start a new subculture. The cultures were nurtured in a growth chamber with a light/dark temperature range of 28–30 °C/20–25 °C and a photoperiod of 16 h at an intensity of 1200 mol m^−2^ s^−1^ from cool-white fluorescent lamps [44]. From 14-day-old Azolla culture, plants were harvested, washed several times with agitation in a large volume of tap water to remove epiphytic microorganisms, then blotted gently with tissue papers.

### 4.3. Phytochemical Screening

#### 4.3.1. Extraction

The fresh Azolla plants were shade-dried until total dryness. The plants were then thoroughly powdered in a high-power blender. Ten grams of powdered samples of *A. caroliniana* or *A. filiculoides* were soaked in 100 mL methanol (80%) in the dark for one week with continuous shaking. A rotary evaporator set to 40 °C was used to concentrate the extracts to a dry consistency. A total yield of 0.540 ± 0.143 g of methanol-free extract was obtained.

#### 4.3.2. Gas Chromatography–Mass Spectrometry Analysis (GC-MS)

Instruments used for the GC-MS analysis included a TRACE GC Ultra Gas Chromatograph (Thermo Scientific Inc., Waltham, MA, USA) and a Thermo mass spectrometer detector (ISQ Single Quadrupole Mass Spectrometer) (Thermo Fisher Scientific—Waltham, MA, USA). A TR-5 MS column (30 m × 0.32 mm i.d., 0.25 μm film thickness) was used in the GC-MS instrument. The following temperature schedule was used to conduct the analyses: helium as the carrier gas at a flow rate of 1.0 mL/min and a split ratio of 1:10:60 °C for 1 min, increasing by 4 °C every minute up to 240 °C, then maintained for 1 min. The injector and the detector were held at a constant 210 °C. Electron ionization (EI) mass spectra were collected at 70 eV in the *m*/*z* 40–450 range. AMDIS software was used to decipher the retention indices (relative to n-alkanes C8–C22), mass spectra (matched to the Wiley spectral library collection and the NSIT lib database), and identity of the chemical contents of the methanolic extract.

### 4.4. Antimicrobial Activity

The well diffusion method was used for preliminary studies of the inhibition zone [45].

#### 4.4.1. Preparation of Fungal *Inocula*

The inoculum was prepared using a 7-day-old culture of each fungus on Sabouraud dextrose agar. Malt broth tubes (4 mL) were inoculated with a 4 mm diameter piece removed from the culture of each fungus. The inoculated agar was poured into malt agar plates.

#### 4.4.2. Preparation of Bacterial *Inocula*

After inoculating Mueller–Hinton broth with a suspension made from colonies grown overnight on an agar plate, using 100 L of the fresh bacterial culture of each bacterial strain in 7 mL soft agar, Mueller–Hinton agar plates were inoculated using a swab that had been submerged in the suspension.

Dimethyl sulfoxide (DMSO) was used to dissolve 10, 5, and 2.5 mg/mL of methanol extracts of *Azolla caroliniana* and *Azolla filiculoides*. Then, 100 µL of each extract solution was added to three wells (well diameter: 6.0 mm) in agar plates with a diameter of 9 cm for each set of tests. Some wells received a negative control solution (DMSO), while others received a positive control solution (reference antifungal and antibacterial medicines). After 24 h at 37 °C (for the studied bacteria) and 27 °C to 32 °C (7–10 days), the inhibitory zone was determined around each well (for tested fungi). The antimicrobial effects of the studied plant extracts were reported as millimeter-wide inhibitory zones (mm).

### 4.5. Antioxidant Activity Using Ferric-Reducing Antioxidant Power (FRAP) Assay

**Figure 8:**
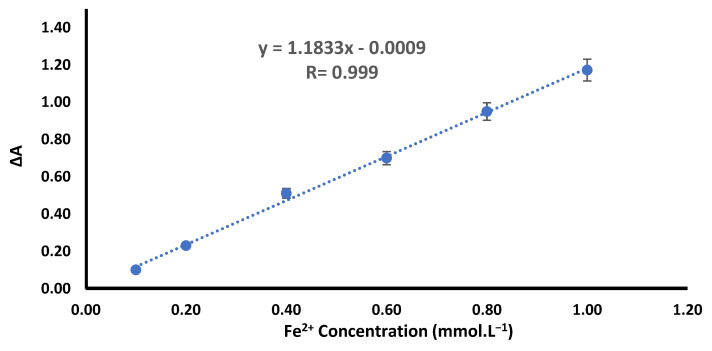
Standard curve of FRAP.

Ferric-reducing antioxidant power (FRAP) assay is a widely used method that involves the use of antioxidants as reductants in a redox-linked colorimetric reaction, wherein Fe^3+^ is reduced to Fe^2+^. This ferric (Fe^3+^)-to-ferrous (Fe^2+^) ion reduction at low pH values causes the formation of a colored ferrous-probe complex from a colorless ferric-probe complex that can be measured at 593 nm. Briefly, 10 μL of the samples was added to each well, plus 190 μL of FRAP assay buffer (10 mmol·L^−1^ of TPTZ, 40 mmol·L^−1^ of HCl, 20 mmol/L FeCl_3_·6H_2_O in 300 mmol·L^−1^ acetate buffer pH 3.6). The absorbance of each well was measured using a SPECTRONIC 200 UV-vis spectrophotometer (Thermo Scientific, Waltham, MA, USA) at 593 nm at 0 and 4 min, and the difference in absorbance (∆A) between the final reading and the reading at zero was computed for each sample and compared to the ∆A of a standard curve (Figure 8) constructed using different concentrations (10–1000 mmol/L) of Fe^2+^ standard solution (FeSO_4_·7H_2_O) tested in parallel with the samples. Using the established procedure, we used logarithmic regression analysis to compare the EC_50_ (half-maximal effective concentration) values derived from the relevant dose–response curves, settling on ascorbic acid as the standard antioxidant. The limit of detection (LOD) and limit of quantification (LOQ) of Fe^2+^ are 7.7 and 8.4 µmL.L^−1^, respectively, theoretically calculated using the following formulas: LOD = (Ybl + 3Sbl)/b and LOQ = (Ybl + 3Sbl)/b, where Ybl is the response obtained in the blank, Sbl is the standard deviation obtained in the blank, and b is the slope of the standard curve.

### 4.6. Anti-Inflammatory Activity

#### 4.6.1. Inhibition of Protein Denaturation

Protein denaturation inhibition was measured according to the method described Mizushima and Kobayashi [46] and Sakat et al. [47] with a minor adjustment. First, 100 μL of plant extract was mixed with 500 μL of bovine serum albumin 1%. After sitting at room temperature for 10 min, this combination was heated to 51 °C for 20 min. Once the solution had cooled to room temperature, its absorbance was measured at 660 nm. Sodium diclofenac was used as a quality reference point. Protein denaturation was inhibited by a percentage determined by experimenting three times:

$$\%\;\mathrm{Inhibition}=\frac{A1-A2}{A1}\times 100$$

where A1 is the absorbance of the blank, and A2 is the absorbance of the sample.

#### 4.6.2. Membrane Stabilization against Heat-Induced Hemolysis

Human blood was obtained from healthy participants, then centrifuged at 3000 rpm for 10 min to remove clots. The red blood cell layer was removed and diluted with phosphate-buffered saline to a final concentration of 10% (*v*/*v*) (PBS). All extracts were diluted to a final volume of 100 μL before being mixed with 100 μL of 10% RBC. After 30 min of heating at 56 °C, the resultant solution was centrifuged at 2500 rpm for 10 min at room temperature to remove any solids. Absorbance was measured at 560 nm after collecting the supernatant. Acetylsalicylic acid was utilized as a positive control, while a blank of 100 μL PBS was used as a negative control to determine the percentage of membrane stability according to Yoo et al. [48] and Sakat et al. [47].

$$\%\;\mathrm{Inhibition}=\frac{A1-A2}{A1}\times 100$$

where A1 is the absorbance of the blank, and A2 is the absorbance of the samples.

#### 4.6.3. Inhibition of Nitric Oxide Production in the RAW 264.7 Macrophage Cell Line

RAW264.7 murine macrophages (ATCC^®^) were grown in a humidified 5% CO_2_ incubator with complete Dulbecco’s Modified Eagle’s Medium (DMEM, Corning, USA) supplemented with 10% fetal bovine serum, penicillin (100 U/mL), streptomycin sulfate (100 g/mL), and 2 mM L-glutamine. To prepare the cells for passaging and treatment, phosphate-buffered saline was used, and the cells were scraped off the flasks with sterile scrapers (SPL, Spain). Overnight, 96-well microwell plates were seeded with RAW 264.7 cell stock (0.5 × 10^6^ cells/mL). The following day, non-induced triplicate wells were filled with the sample vehicle-containing media (DMSO, 0.1%, by volume). Inflammation was induced by administering lipopolysaccharide (LPS) at 100 ng/mL in full culture conditions to a set of triplicate wells. Sample groups of triplicate wells received increasing concentrations of the dissolved extract (6.25%, 12.5%, 25%, 50%, and 100 g/mL; final concentration of DMSO = 0.1% by volume) were added to culture media containing LPS. Nω-nitro-L-arginine methyl ester hydrochloride (L-NAME, 1 mM) was a successful anti-inflammatory control. Following a 24 h incubation period, a Griess assay [48] determined that all wells had NO end products. To create colored diazonium salt, equal amounts of culture supernatants and Griess reagent were combined, incubated at room temperature for 10 min, and read at an absorbance of 540 nm on a Tecan Sunrise™ microplate reader (Austria). Using an Alamar Blue™ reduction assay to measure cell viability, the NO inhibition percent of the test extract was computed relative to the LPS-induced inflammation group [49]. Using the non-linear regression analysis function of GraphPad Prism v9.0.2 software (San Diego, CA, USA),.the IC_50_ of inhibition was determined.

### 4.7. Cytotoxicity Activity against the HepG2 Cell Line

Nawah Scientific Inc. (Mokatam, Cairo, Egypt) supplied the HepG2 hepatocellular carcinoma cells used in this study. At 37 °C in a humidified 5% (*v*/*v*) CO_2_ atmosphere, cells were grown in DMEM media containing 100 mg/mL of streptomycin, 100 units/mL of penicillin, and 10% heat-inactivated fetal bovine serum. A sulforhodamine B (SRB) cell cytotoxicity experiment was performed to measure cell viability and drug cytotoxicity. The IC_50_ assay is quick, easy, reproducible, and sensitive [50,51]. In brief, 96-well plates were seeded with aliquots of 100 μL cell suspension (5 × 103) and cultured in full medium for 24 h. Another aliquot of 100 μL medium containing medicines at concentrations ranging from 0 to 1600 mg/mL of both extracts or doxorubicin (0 to 100 mg/mL) was used to treat the cells. After 72 h of drug exposure, the medium was changed out for 150 μL of 10% TCA, and the cells were incubated at 4 °C for an additional hour to fix them. After removing the TCA solution, the cells were rinsed with distilled water five times. A total of 70 μL of a 0.4% *w*/*v* SRB solution was added, and the mixture was allowed to stand at room temperature in the dark for 10 min. The plates were cleaned three times with 1% acetic acid, followed by overnight air drying. The protein-bound SRB stain was dissolved by adding 150 μL of TRIS (10 mM), and absorbance was read at 540 nm on a BMGLABTECH^®^-FLUOstar Omega microplate reader (Ortenberg, Germany) [50,51].

### 4.8. Statistical Analysis

Data are presented as means ± standard deviation, and the experiments were conducted in triplicate to ensure accuracy and reliability. Statistical analysis was performed using SPSS v.18.0 software. For the analysis, either a *t*-test or one-way ANOVA was employed, with the significance level set at *p* < 0.05. 

## 5. Conclusions

Our data indicate that the methanolic extracts of *A. caroliniana* and *A. filiculoides* contain differential ingredients and possess antioxidant, anti-inflammatory, and HepG2 cytotoxic activity. Furthermore, these extracts have antimicrobial activity against *Geotrichum candidum*, *Enterococcus faecalis*, and *Klebsiella pneumonia*. Therefore, we suggest that *A. caroliniana* and *A. filiculoides* can be used as food supplements and supportive therapeutic agents.

## Figures and Tables

**Table 1:** Phytochemical substances found in the methanolic extract of *A. caroliniana* (C) and *A. filiculoides* (F) by GC-MS.

Rt	Compound	M.F	M.W.	Peak Area (%)	
				C	F
3.03	9-Octadecenoic acid, (2-phenyl-1,3-dioxolan-4-yl) methyl ester, cis-	C_28_H_44_O_4_	444.6	3.03	1.34
11.27	Isochiapin B	C_19_H_22_O_6_	346	-	0.74
12.51	Dimethyl lauramine	C_14_H_31_N	213	13.11	23.89
15.94	Dimethyl myristamine	C_16_H_35_N	241	5.98	9.12
19.56	Methyl palmitate	C_17_H_34_O_2_	270	4.57	3.14
20.33	Pentadecanoic acid	C_15_H_30_O_2_	330	2.36	2. 90
22.22	Ethyl linoleate	C_20_H_36_O_2_	308	2.26	1.14
22.33	9,12-Octadecadienoic acid (Z,Z)-, 2-hydroxy-1-(hydroxymethyl)ethyl ester	C_21_H_38_O_4_	354	5.16	3.02
22.54	N-Methyl-N-benzyltetradecanamine	C_22_H_39_N	317	5.85	9.78
22.73	Cyclopropanebutanoic acid, 2-[[2-[[2-[(2-pentylcyclopropyl)meth yl]cyclopropyl]methyl]cyclopropyl] methyl]-, methyl ester	C_25_H_42_O_2_	374	0.53	-
23.11	Tricyclo [20.8.0.0(7,16)]triacontane, 1(22),7(16)-diepoxy-	C_30_H_52_O_2_	444	2.93	2.07
25.57	Calcitriol	C_27_H_44_O_3_	416	1.81	2.64
34.27	Nicotiflorin	C_27_H_30_O_15_	594	2.92	1.41
34.65	Trilinolein	C_57_H_98_O_6_	878	1.28	2.10
36.49	Rhodopin	C_40_H_58_O	554	10.65	12.05

Rt: retention time (in minutes); MF: molecular formula; MW: molecular weight.

**Table 2:** Mean zone of inhibition (mm) of the methanol extracts of *A. caroliniana* (C) and *A. fliliculoides* (F) produced on pathogenic microorganisms.

	Reference Drug	C	F
Fungi	Ketoconazole (100 μg/mL)		
*Fusarium oxysporum* (RCMB 001004)	19 ± 4.00	NA	NA
*Trichophyton rubrum* (RCMB 025002)	22 ± 2.31	NA	NA
*Candida albicans* (RCMB 005003)	20 ± 2.38	NA	NA
*Geotrichum candidum* (RCMB 041001)	26 ± 3.19	15 * ± 2.44	12 * ± 2.69
Gram-positive bacteria:	Gentamycin (4 μg/mL)		
*Staphylococcus aureus* ATCC 25923	24 ± 3.19	NA	NA
*Enterococcus faecalis* ATCC 29212	26 ± 4.16	12 * ± 1.34	10 * ± 2.14
Gram-negative bacteria:	Gentamycin (4 μg/mL)		
*Enterobacter cloacae* ATCC 23355	30 ± 2.00	NA	NA
*Klebsiella pneumonia* ATCC 13883	21 ± 1.96	11 * ± 1.36	13 * ± 2.91

NA: No activity. Data are reported as means of three replicates ± SD; * significant difference compared with the reference drug according to ANOVA at *p* < 0.05.

**Table 3:** The antimicrobial activity of the methanol extracts of *A. caroliniana* (C) and *A. fliliculoides* (F) as the minimum inhibitory concentration produced on pathogenic microorganisms.

Test Organism	Minimum Inhibitory Concentration (MIC, μg/mL)	
	C	F
Fungi		
*Fusarium oxysporum* (RCMB 001004)	NA	NA
*Trichophyton rubrum* (RCMB 025002)	NA	NA
*Candida albicans* (RCMB 005003)	NA	NA
*Geotrichum candidum* (RCMB 041001)	312.5 ^a^ ± 7.5	625 ^b^ ± 4.1
Gram-positive bacteria:		
*Staphylococcus aureus* ATCC 25923	NA	NA
*Enterococcus faecalis* ATCC 29212	625 ^a^ ± 3.2	1250 ^b^ ± 11.8
Gram-negative bacteria:		
*Enterobacter cloacae* ATCC 23355	NA	NA
*Klebsiella pneumonia* ATCC 13883	1250 ^a^ ± 9.6	312.5 ^b^ ± 5.8

Data are presented as the means of three replicates (*n* = 3) ± standard error. NA: No activity. Values not sharing a common superscript letter differ significantly at *p* < 0.05.

**Table 4:** IC_50_ (µg/mL) of the methanolic extracts of *A. caroliniana* and *filiculoides* in FRAP, inhibition of protein denaturation, inhibition of heat-induced hemolysis, inhibition of NO production, and cytotoxicity against the HepG2 cell line.

Biological Activity	Reference Drug	*A. caroliniana*	*A. filiculoides*
FRAP	(Ascorbic acid) 416.21	55.86	92.78
Inhibition of protein denaturation	(Sodium diclofenac) 218.6	360.0	527.3
Inhibition of heat-induced hemolysis	(Acetyl salicylic acid) 68.25	42.78	45.85
Inhibition of NO production	-	33.92	23.8
Cytotoxicity against the HepG2 cell line	(Doxorubicin) 0.303	216.86	421.20

## Data Availability

Upon request.

## Abbreviations

A, Azolla; GC-MS, gas chromatography–mass spectrometry; FRAP, ferric-reducing activity power; RBCs, red blood cells; NO, nitric oxide; HepG2, human hepatocellular carcinoma cell line; C, Caroliniana; F, Filiculoides; MIC, minimum inhibitory concentration; NA, no activity; IC_50_, half-maximal inhibitory concentration; RAW 264.7, macrophage cell line; LPS, lipopolysaccharide; IL-10, interleukin-10; iNOS, inducible nitric oxide synthase; COX-2, cyclooxygenase-2; NF-κB, nuclear factor-kappa B; TNF-α, tumor necrosis factor alpha; IL-1β, interleukin-1beta; IL-6, interleukin-6; COPD, chronic obstructive pulmonary disease; HO-1, heme oxygenase-1; Si, small interfering; RNA, ribonucleic acid; HCC, hepatocellular carcinoma; KCL, potassium chloride; CaCl_2_, calcium chloride; KNO_3_, potassium nitrate; Ca(NO_3_)_2_, calcium nitrate; NCCLS, National Committee for Clinical Laboratory Standards; EI, electron ionization; AMDIS software, software for GC-MS data interpretation from NIST (DMSO, dimethyl sulfoxide; FeIII–TPTZ, ferric-tripyridyltriazine; FeII, Ferrous; TPTZ, tripyridyltriazine; FeCl_3_·6H_2_O, Ferric chloride hexahydrate; FeSO_4_·7H_2_O, ferrous sulfate heptahydrate; ∆A, difference in absorbance; EC_50_, half-maximal effective concentration; PBS, phosphate-buffered saline; DMEM, Dulbecco’s Modified Eagle’s Medium; SRB, sulforhodamine B; TCA, trichloroacetic acid.
